# Fabrication and Surface Interactions of Super-Hydrophobic Silicon Carbide for Membrane Distillation

**DOI:** 10.3390/nano9081159

**Published:** 2019-08-13

**Authors:** Vittorio Boffa, Cristian Lunghi, Cejna A. Quist-Jensen, Giuliana Magnacca, Paola Calza

**Affiliations:** 1 Center for Membrane Technology, Department of Chemistry and Bioscience, Aalborg University, Fredrik Bajers Vej 7H, 9220 Aalborg Øst, Denmark; 2 Dipartimento di Chimica, Universitá di Torino, Via P. Giuria 7, 10125 Torino, Italy; 3 NIS Interdepartmental Center, Universitá di Torino, Via P. Giuria 7, 10125 Torino, Italy

**Keywords:** isosteric heat, low-energy surface, fluorocarbon, desalination, water detoxification

## Abstract

Hydrophilic silicon carbide was modified by surface deposition of a super-hydrophobic coating that is based on perfluorosilanes. The modification was proven to yield membrane surfaces with contact angles that were higher than 145° and to be stable under hydrothermal conditions. The measurement of the isosteric heat of adsorption of water and toluene by microgravimetry showed that, after modification, the membrane material was fully covered by a low-energy surface, which is consistent with the fluorocarbon moieties that were introduced by the modification. The same modification method was applied to a commercial multichannel SiC membrane tube (nominal pore size = 0.04 µm), which was tested in a direct contact membrane distillation apparatus. The membrane was permeable to water vapour and volatiles, but it showed full rejection for salt ions and organic pollutants with low vapour pressure (such as ibuprofen and caffeine). Moreover, the membrane was reusable, and its performances were stable with no sign of pore wetting over 8 h of filtration.

## 1. Introduction

**Figure 1:**
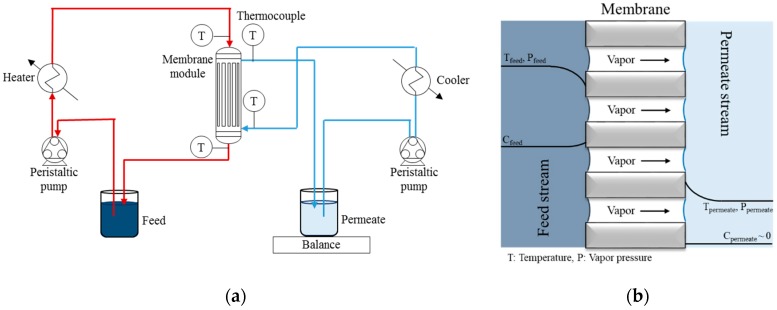
(**a**) Scheme of a typical direct contact membrane distillation (MD) apparatus and (**b**) cross-sectional diagram of a MD action: vapour pressure (p) temperature (T), and concentration of solutes (C) at the feed and the permeate side.

Membrane distillation (MD) is an emerging separation technology that combines the advantages of thermal distillation and membrane filtration [1,2,3]. As shown in Figure 1, in this process a mesoporous or macroporous hydrophobic membrane acts as a barrier between a hot feed solution and the cold permeate. The membrane is not permeable to water in the liquid state due to its hydrophobic surface, but it allows for the permeation of water molecules in the form of vapour. A vapour pressure gradient is established between the hot feed side and the cold permeate side. Consequently, a flux of steam is generated across the membrane and it is condensed on the permeate side of the membrane. Only water molecules and volatiles can permeate the membrane, which on the contrary retains dissolved salts and non-volatile molecules. Hence, this type of filtration presents several advantages over traditional processes, as: (i) the ability to operate at a lower operating feed temperature than conventional distillation, which makes it possible to exploit low-grade heat streams; (ii) a much lower hydrostatic pressure than the traditional nanofiltration and reverse osmosis processes, thus allowing for simpler and cheaper membrane modules; and, (iii) permeability and selectivity are both negligibly affected by the increase of osmotic pressure during filtration, allowing for achieving high salt concentration on the feed side. In this way, the volume of brine discharged into the environment is reduced, and it is possible to recover minerals from the concentrate by crystallization [4,5,6].

Current pilot and demonstrative MD installations rely on commercial polymeric (e.g., polypropylene) membranes [7,8,9,10]. However, these polymeric membranes have some important drawbacks, which include the low thermal stability and low chemical resistance, which implied difficulties in their cleaning and short service time. Ceramic membranes are more robust than their polymeric counterpart, but they have rarely been considered for this application due to their intrinsic hydrophilicity. Indeed, in this application material design should prevent membrane wetting. This occurs when the water at the liquid state penetrates into the membrane pores and transports solutes across the membrane, thus compromising its selectivity. In recent years, a few attempts have been made to fabricate durable ceramic membranes for MD [11,12,13,14,15,16,17,18,19,20,21,22,23,24,25]. The common approach is based on the alteration of the surface properties of the ceramic material from hydrophilic to hydrophobic by the chemical reaction of surface hydroxyls with fluorinated silanes [26]. Such membranes have been demonstrated to function well under MD conditions and to be re-usable, thus providing an alternative to the less robust traditional polymeric membranes. Alternative methods involve the reaction of surface hydroxyls with silicone oil at a higher temperature and interfacial polymerization [27]. These modification procedures should be designed for complete functionalization of large area membrane modules in order for ceramic MD membranes to achieve real-scale application, and the fluorosilane layer should be able to withstand long operation time without being washed out. Indeed, the wetting of even a small fraction of the membrane pores can compromise the selectivity of the entire membrane module.

In this work, we present the fabrication of a new super-hydrophobic silicon carbide (SiC) membrane and report its application in MD. This membrane was prepared by surface deposition of a fluorinate silsesquioxane gel and further curing with perfluorooctyltrichlorosilane. This approach was used in order to achieve complete coverage of the membrane surface with a super-hydrophobic coating. To the best of our knowledge, for the first time, microgravimetric analyses of the adsorption of water and toluene vapours on membrane powder samples were performed, thus providing new insight on the surface characteristics of MD materials and on their interaction with water and organic vapours. The membrane was eventually tested in a direct contact membrane distillation (DCMD) apparatus reflecting good performances in the desalination and detoxification of model water solutions.

## 2. Experimental Part

### 2.1. Chemicals and Materials

The following chemicals were purchased from Sigma-Aldrich (St. Louis, MO, USA): tetraethyl orthosilicate (purity = 98%, acronym = TEOS), 1H,1H,2H,2H-Perfluorodecyltriethoxysilane (98%, PFDS), 1*H*,1*H*,2*H*,2*H*-perfluorooctyltrichlorosilane (97%, PFCS), acetonitrile (99.8%) nitric acid (69%), potassium bromide (99.9%), ethanol (99%), toluene (99.9%), ibuprofen (98%), 2,4-diclorofenolo, (99%), caffeine (anhydrous 99%), and sodium chloride (99.5%). Monopotassium phosphate (anhydrous, 99%) was purchased from Merck (Burlington, MA, USA). The model solutions for the filtration experiments were prepared with MilliQ water.

Depending on the development stage, in this work modified silicon carbide was used as powder, as flat-sheet supported membrane, and as multi-channel membrane tube were tested. A commercially available α-SiC powder (NF25, ESK-SIC GmbH, Frechen, Germany), with an average particle size *d*_50_ = 0.4 μm, was used as unsupported membrane material. A SiC ultrafiltration (nominal pore size ~0.04 μm) multichannel tube was acquired from Liqtech International A/S (Ballerup, Denmark) and used to fabricate a hydrophobic MD membrane. It has external diameter of 25.4 mm, length of 305 ± 1 mm, and presents 31 open channels (Ø = 3 mm). The fabrication procedure was optimized on flat sheet SiC membranes (20 × 10 mm^2^), which were also provided by Liqtech International and consisted of the same multilayer structure of the membrane tube. Before modification, the powdered SiC samples were pre-treated according to the same temperature programme and in the same atmosphere used for the fabrication of the top-layer on the flat and tubular membranes.

### 2.2. Surface Modification

SiC powder, flat-sheet samples, and the tube membrane were modified according to the same two-step procedure. In the first step, the surface of the samples was coated with a fluorinated polyhedral oligomeric silsesquioxane gel (F-POSS) [28], which was synthetized from a mixture of TEOS:PFDS:ethanol, with the following molar ratio: 1:0.0113:7.19. After stirring this mixture for 20 min at room temperature (r.t.), 0.01 M nitric acid was added dropwise (0.80 mL for 1 mL of TEOS). In this way, we provided the mixture with water for hydrolysis and with acid as catalyst. The mixture was stirred for 3.5 h to allow for F-POSS formation. Subsequently, the solution was kept at 4 °C to prevent particle growth and gelation until use for membrane modification. The SiC samples (powder, flat sheet membranes, and the multichannel tube) were immersed in the F-POSS solution for 10 min at r.t., then, the samples were washed three times by immersion in ethanol (1 min for each time). F-POSS condensation and adhesion on the support was promoted by curing the samples at 200 °C for four hours under argon flux. In the second step, the samples were immersed in a 0.1% V/V solution of PFCS in hexane for 30 min. Afterwards, the samples were removed from the solution, dried in air, and annealed at 200 °C for 4 h under argon flux.

### 2.3. Material Characterization

The apparent contact angle (CA) was used to estimate the hydrophobicity of the ceramic membrane before and after surface modification and to verify that they did not lose their hydrophobic character while being contacted with hot water. CA was measured by means of a OnePlus 6 camera (Sony IMX 519 image sensor, Tokyo, Japan). The morphology of the ceramic membrane was observed by Scanning Electron Microscope (SEM, FEI NOVA NanoSEM 230, Thermo Fisher Scientific, Hillsboro, OR, USA). Fourier Transform Infrared Spectroscopy (FT-IR) spectra were acquired on an IRTracer−100 (IRTracer-100, Shimadzu, Shanghai, China) in the transmission mode on KBr suspension. Microgravimetric water and toluene adsorption isotherms were obtained at various temperatures with a microbalance apparatus (IGA002 by Hiden, Warrington, UK), contacting the powders (about 100 mg) with water and toluene vapours. The temperature control was guaranteed by a thermostatic water bath.

### 2.4. DCMD Experiments

After modification, the tubular membrane was fixed in a Plexiglas housing with epoxy resin and then tested in the direct contact membrane distillation apparatus (Figure 1a), which consists of a feed tank (with the water to be treated) and a permeate tank (with MilliQ water). The temperatures of the feed and permeate streams were adjusted to around 66 and 21 °C, respectively, which were controlled by a Thermo Haake^®^ K20 bath and a Julabo FP50-HL by two heat exchangers. The cross-flow velocity of the two solutions was kept at 30 L/h in counter-current mode by a Masterflex thermostatic pump. The permeation rate (transmembrane flux) was measured by the weight increase in the permeate tank.

The fabricated membrane module was tested with different feed solutions, including high concentrated saline solution (5 wt% NaCl) and solutions of model organic pollutants (ibuprofen, caffeine, and 2,4-dichlorophenol—all with initial concentration of 5 mg L^−1^). Salt and pollutants rejections (%) were calculated by Equation (1), where *V_p_*^0^ is the starting volume at the permeate side; while *V_p_*, *C_p_*, and *C_f_* are, respectively, the permeate volume, the permeate concentration, and the feed concentration during filtration. NaCl concentration was determined by measuring the sample conductivity with a Mettler Toledo S47K. Simultaneous determination of the concentrations of the organic pollutants was attained by High Performance Liquid Chromatography (HPLC, Dionex with Chromeleon 6.80 software) with a Luna^®^ 5 μm C18 100 Å column (Phenomenex), 250 × 4.60 mm. Under these experimental conditions, the detection limits for three pollutants were <0.05 mg/L.

(1) $$\mathrm{Rejection}\left(\%\right)=\left(1-\frac{C_{p}\frac{V_{p}}{V_{p}-V_{p}^{o}}}{C_{f}}\right)\times 100$$

## 3. Results and Discussion

### 3.1. Surface Modification

**Figure 2:**
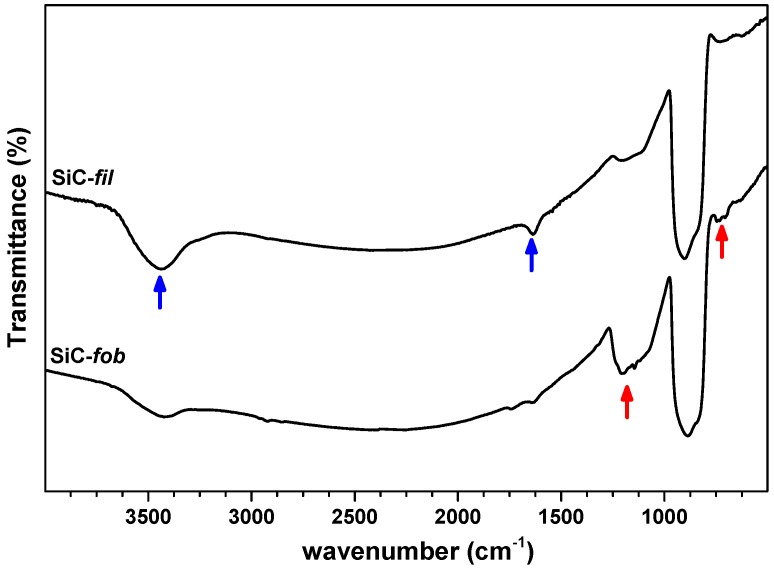
FT-IR spectra of the membrane material, before (SiC-*fil*) and after modification (SiC-*fob*). The blue arrows indicate the signals typical of the hydrated layer at the SiC-*fil* surface, whereas the red arrows evidence the signals typical of the C–F vibrations in the SiC-*fob* spectrum.

In this work, silicon carbide was made a suitable material for membrane distillation by a fluorocarbon coating, as described in the experimental section. At first, the surface modification procedure was investigated and then confirmed by FT-IR analysis on powder samples. The absorption spectrum of the powdered membranes before modification (SiC-*fil*) shows weak bands between 500 and 700 cm^−1^ and a more intense and complex absorption between 750 and 950 cm^−1^, which can be ascribed to the characteristic stretching vibrations of the Si–C bond [29]. The third absorption characterised by a complex shape present in the interval 1000−1250 cm^−1^ arises from the contribution of different vibrations, one of which is the Si–O–Si vibration (that is typically centred at 1100 cm^−1^), which can be ascribed to the native oxide layer, which covers the SiC particles. Furthermore, the SiC-*fil* spectrum shows the typical peaks of H–O–H bending and O–H stretching (blue arrows in Figure 2), which evidence the presence of adsorbed water molecules and silanol groups, respectively. Therefore, the FT-IR analysis confirms the presence of a hydrophilic oxide surface layer [30], which is prone to reaction with the fluorinate coating.

After modification (SiC-*fob* sample), the FT-IR spectrum shows new absorption signals in the region between 680 and 760 cm^−1^ and between 1050 and 1250 cm^−1^, which are characteristic of the fluorocarbon compounds [31,32] (red arrows in Figure 2). These new bands are weak in intensity, as the silanisation reaction only interests the surface of the material (where Si–OH groups were available for the functionalisation reaction) and arise from a range of different CF_2_ and CF_3_ moieties. As a consequence, the intensity of the peak that is related to the presence of Si–OH groups (above 3000 cm^−1^) decreases because of their consumption during the functionalisation reaction, and the intensity of the band that is related to adsorbed water molecules (1630 cm^−1^) decreases for the modified hydrophilicity of the material surface.

The modification procedure was repeated on the flat-sheet membrane samples. The atomic composition of the membrane surface, as measured by Energy-dispersive X-ray spectroscopy (EDX), is reported in Table 1. EDX data confirm the results of FT-IR spectroscopy: no fluoride atoms were detected in SiC-*fil*, while the fluoride concentration in SiC-*fil* is 1.5 atom%. The presence of fluoride atoms on the surface of the SiC‑*fob* membrane is a direct consequence of the surface modification: the low F concentration is consistent with the fact that the chemical modification only occurs at the surface of the material. The hydrophilicity of the membranes was determined by measuring the contact angle of the water droplets on the surface of flat-sheet SiC membranes. Such a measurement cannot be easily performed on the SiC-*fil* membrane, because this highly porous and hydrophilic material quickly drains water. Therefore, the measurement for this material was taken on a wet surface, yielding a contact angle of 31.5° ± 3.7°, which corresponds to a hydrophilic surface. The hydrophilic nature of the SiC-*fil* surface is related to the presence of external surface SiO_2_ layer with its characteristic hydroxyl groups being revealed by FT-IR analysis [30]. On the contrary, a contact angle of 143.2° ± 0.5° was measured after modification (SiC-*fob*) under MD conditions. Such hydrophobic surface prevented the liquid water to penetrate in the membrane pores, thus suggesting SiC-*fob* to be suitable for MD systems.

**Figure 3:**
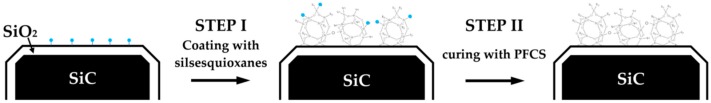
Membrane modification: condensation of the silsesquioxane gel with surface silanols (STEP I), and reaction of the remaining hydroxyls with 1*H*,1*H*,2*H*,2*H*-perfluorooctyltrichlorosilane, PFCS (STEP II). Blue dots indicate OH groups.

A scheme of the chemical modification of the membrane surface, according to the FT-IR, EDX, and contact angle measurements is reported in Figure 3. The silsesquioxane gel is anchored on the membrane surface by condensation of silanol groups. Subsequently, the remaining silanols react with PFCS, yielding a fully hydrophobic surface.

In order to investigate the stability of the coating, SiC-*fob* samples were soaked in boiling water for one day or three days. After this treatment the water contact angle did not decrease, on the contrary, it increased to 146.8° ± 0.7° after one day and 148.9° ± 1.4° after three days. Therefore, after soaking, the surface of the membrane material becomes super-hydrophobic by definition, as the measured water contact angle is higher than 145° [33]. These results clearly indicate that the hydrophobic coating is stable. The small increase in the contact angle can be explained by considering the condensation of the residual isolated silanol groups, which typically occurs in silica gels under hydrothermal exposure.

### 3.2. Adsorption of Vapours

**Figure 4:**
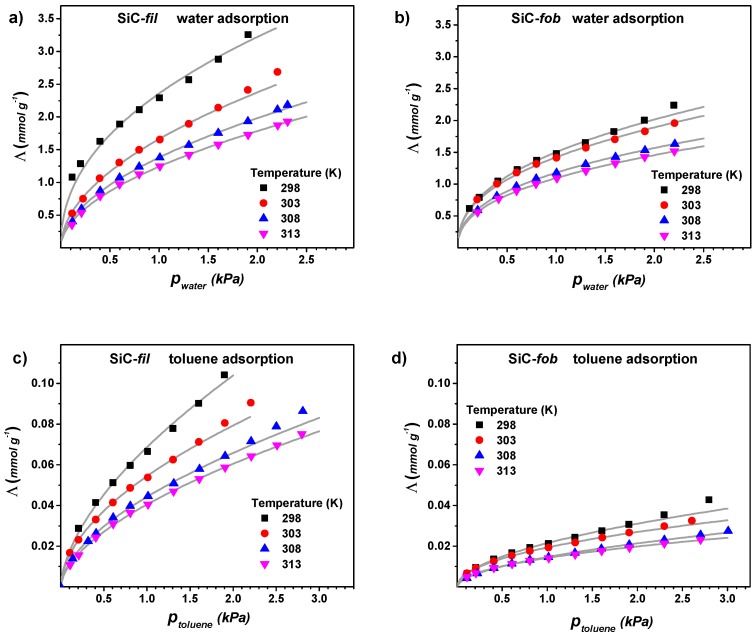
Microgravimetric isotherms for the adsorption of water (**a**,**b**) and toluene (**c**,**d**) on SiC-*fil* and SiC-*fob* at 298, 303, 308, and 313 K. Grey lines represent the power fitting of vapour uptake (*Λ*) data by the a Freundlich-type power function (Λ=Λ1KPap1n).

We decided to perform a microgravimetric study of the adsorption of water and organic vapour on the unmodified (SiC-*fil*) and modified (SiC-*fob*) SiC material, as it is hard to investigate the hydrophilicity of highly porous and rough materials by simple contact angle measurements. Hence, powder samples were contacted with vapours of water and toluene. The adsorbed amount at the equilibrium (*Λ*) is plotted in Figure 4 as a function of vapour pressure. These measurements were performed at four temperatures in the range between 298 and 318 K, with the aim of calculating the isosteric heat of adsorption for the different solid-adsorbate systems. The adsorption cycle was repeated twice after outgassing the sample under vacuum at the same temperature that was adopted for adsorption in order to check the reproducibility of the measurement and the eventual presence of irreversible adsorption. In all cases, the adsorption results are completely reversible and reproducible. For this reason, only the curve relative to the second adsorption run are reported in the following. A power function provided a better fitting of the adsorption isotherms than the Langmuir and Sips models, especially for the membrane material before modification. This is not surprising while considering that this material has a heterogeneous surface (partially oxidized in form of a SiO_2_ layer) [30], and it cannot be well described by the above-mentioned models.

**Figure 5:**
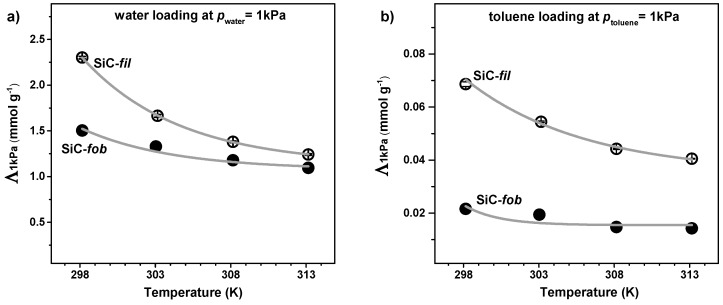
Uptake at 1kPa(*Λ_1kPa_*) of (**a**) water and (**b**) toluene for the membrane materials before and after modification (SiC-*fil* and SiC-*fob*, respectively) in the temperature range between 298 and 313 K.

Both membrane samples (before and after modification) show higher adsorption for water than for toluene. However, at a certain vapour pressure, SiC-*fil* can adsorb higher amounts of water and toluene than SiC-*fob.* Figure 5 shows the different absorption ability of the membrane material before and after modification, where the amounts of vapour adsorbed when the membrane samples were at the equilibrium with 1 kPa of water or toluene (*Λ_1kPa_*) are plotted as a function of the temperature. At this vapour pressure, the molar uptake of SiC-*fil* is about 30 times higher water than for toluene. Moreover, the *Λ_1kPa_* values are always higher for SiC-*fil* than for SiC-*fob*.

The isosteric heat of adsorption (*q*) was calculated by employing the Clausius–Clapeyron equation, that is, by determining the slope of the linear fit for ln*p* versus 1/RT at fixed vapor uptake (*Λ*), with *p* being the equilibrium vapour pressure at a given *T*:

(2) $$q_{\Lambda }=R{\left\{\left[\partial \ln p\right]/\left[\partial \left(1/T\right)\right]\right\}}_{\Lambda }$$

**Figure 6:**
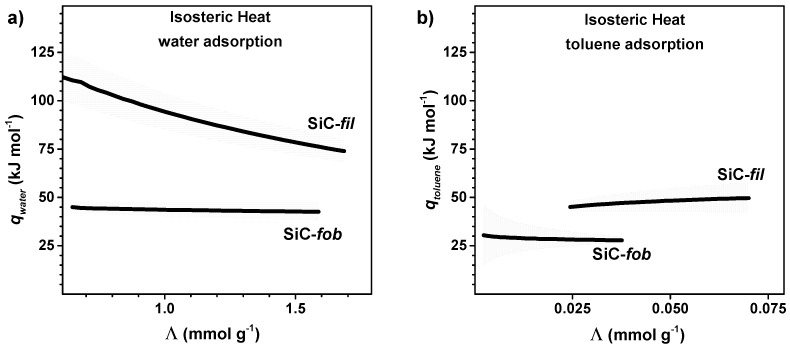
Variation of isosteric heat of adsorption (*q*) with surface coverage (*Λ*) for the adsorption of (**a**) water and (**b**) toluene on SiC-*fil* and SiC-*fob*; *q* values were calculated by fitting Equation (2) on data in Figure 2, Grays bands indicate error from the fitting.

Figure 6 shows the variation of the heat of adsorption of water and toluene as a function of the coverage of the surface of the membrane materials. In the case of SiC-*fil* the isosteric heat of adsorption of water, *q_water_*, drops from 110 ± 12 KJ mol^−1^ at *Λ* = 0.6 mmol g^−1^ to 74 ± 5 kJ mol^−1^ at *Λ* = 1.7 mmol g^−1^. This trend is consistent with a hydrophilic heterogeneous surface, which involves interactions with various sites of different energy. At first, water molecules adsorb on the sites with the highest energy, then on the sites with progressively lower energy. On the contrary, water adsorption on SiC-*fob* shows no big variation of energy in this *Λ* interval, with an average of 43 ± 4 kJ mol^−1^, which suggests weak interactions between the surface and adsorbed water molecules, since this energy is not significantly different from the enthalpy of evaporation of non-adsorbed water, that is 43.3 kJ mol^−1^ [34]. The isosteric heats of adsorption of toluene over SiC-*fil* and SiC-*fob* (*q_toluene_*, as calculated from our adsorption data) are lower than the corresponding *q_water_* (Figure 6) These results are not surprising, as the water molecules can strongly interact with silanol groups present at the surface of SiC-*fil* material via hydrogen bonding, while the interaction with toluene molecules can involve only weaker interactions. Nevertheless, the interaction of toluene vapours with the SiC-*fil* system is stronger (45 ± 3 kJ mol^−1^ and 50 ± 8 kJ mol^−1^) with respect to SiC-*fob* (average = 28 ± 6 KJ mol^−1^). This difference can be explained while considering that the non-treated surface establishes with toluene molecules both dispersion forces through the π-system of the adsorbate and polarization-based interactions that were created by the Si-OH groups, whereas only dispersion forces are present on the hydrophobized surface. Therefore, the heat of adsorption shown by SiC-*fob* is not significantly different from the enthalpy of evaporation of non-adsorbed toluene 33.2 KJ mol^−1^ [34]. In summary, the sorption data indicates that the starting membrane material has a heterogeneous and hydrophilic surface that can have various types of interaction with water and toluene molecules. After modification, the surface of the materials only forms extremely weak interaction with the adsorbates. This indicates that the surface of the membrane material is fully covered by a low surface energy coating, consistently with the fluorocarbon moieties that are introduced by our modification method.

### 3.3. DCMD Tests

**Figure 7:**
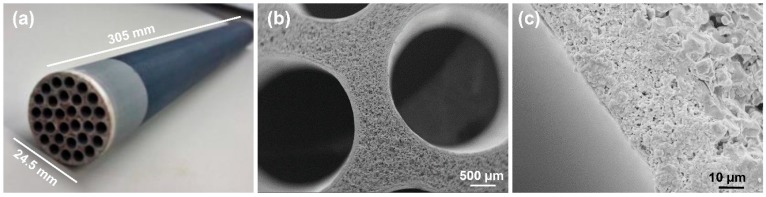
Silicon carbide tube (nominal pore size 0.04 µm) used for preparing the super-hydrophobic MD membrane SiC-fob (**a**) and SEM magnification of its channels (**b**) and multi-layer structure (**c**).

The above reported modification procedure can be easily applied to the membrane tubes. In the Supporting Information, the reader can observe the behaviour of few drops of water on a SiC membrane monotube before (SiC_fil_movie) and after surface modification (SiC_fob_movie). Hence, the multichannel tube that is shown in Figure 7 was modified to obtain a SiC-*fob* membrane. This membrane was soaked for one day in boiling water to washout unreacted modifiers and to achieve a super-hydrophobic surface. Subsequently, it was tested in a direct contact membrane distillation (DCMD) unit for the production of pure water from solutions with high salinity or contaminated with model organic pollutants.

**Figure 8:**
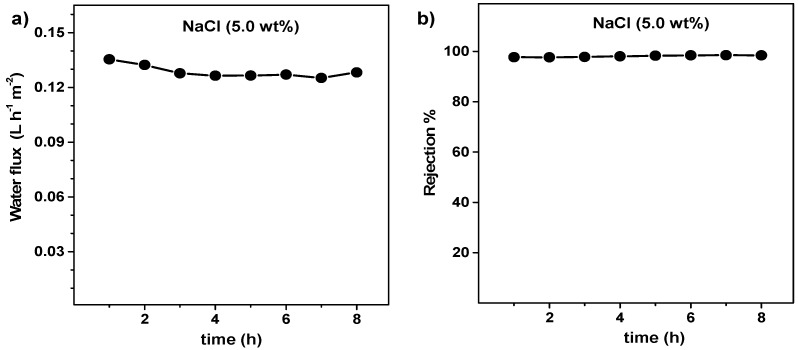
(**a**) permeate flux and (**b**) rejection (%) of the SiC-fob membrane while filtering 50 g L^−1^ NaCl in deionized water over 8 h, rejection was calculated by Equation (1) (feed inlet = 73 °C, feed outlet = 59 °C, permeate inlet = 11 °C, permeate outlet = 31 °C).

The fabricated superhydrophobic membrane was tested while using a simulated brine solution of 5.0 wt% NaCl in deionized water in order to evaluate its stability at extreme conditions. Figure 8 shows the results concerning the filtration. The rejection is remarkably higher than 98% over the 8 h test and no signs of pore wetting were observed, being also the permete flux stable during filtration (0.13 L h^−1^ m^−2^). The flux is still relatively low when compared to the polymeric membranes, but it can be ascribed to the low nominal pore size (0.04 µm) of the virgin SiC membrane tube, and to its multichannel structure. The pore size of membrane distillation membranes is normally in the range of 0.15–0.3 µm [35]. Moreover, reducing the thickness of the channel walls or applying vacuum membrane distillation instead of DCMD will also positively influence the flux. Nevertheless, the hydrophobic coating appears to be effective and stable when being applied to a real-scale membrane module and when operated under realistic DCMD conditions while using high by concentrated saline solutions.

**Figure 9:**
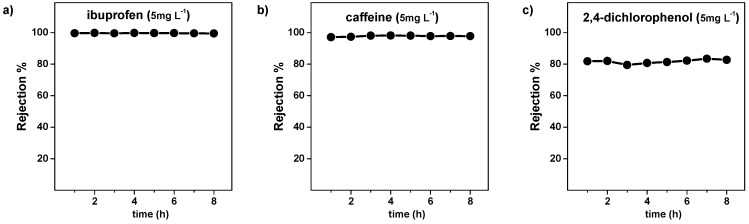
Rejection% of the SiC-fob membrane for model organic contaminants, namely (**a**) ibuprofen, (**b**) caffeine, and (**c**) 2,4-dichlorophenol. The starting concentration on the feed tank was 5 mg L^−1^ for all the pollutants, rejection was calculated by Equation (1) (feed inlet = 73 °C, feed outlet = 59 °C, permeate inlet = 11 °C, permeate outlet = 31 °C).

Thus, the ability of the membrane to retain water pollutants was also investagated. Figure 9 depicts the rejection of the membrane towards ibuprofen, caffeine, and 2,4 dichlorophenol. Again, membrane rejection does not vary during the 8 h duration of the filtration test. However, while complete rejection was observed for ibuprofen and caffeine, the rejection of 2,4 dichlorophenol was only 82% ± 1%. These results can be ascribed to the limits of the membrane distillation process, which is highly effective in the retention of dissolved ions and non-volatile compounds, but it allows permeation of water and volatile organics. Indeed, 2,4 dichlorophenol is the most volatile pollutant that was analyzed in this study. The vapour pressures (at 25 °C) of ibuprofen, caffeine, and 2,4 dichlorophenol are 6 × 10^−3^ Pa, 6 ×10^−4^ Pa, and 1.2 × 10^3^ Pa, respectively.

## 4. Conclusions

In this work, we presented an effective method for the fabrication of ceramic MD membranes. The method was shown to function on powder, on flat-sheet module, and on multichannel membrane tubes. After modification, the membrane presented a super-hydrophobic surface and it was not permeable to liquid water, in the absence of a pressure gradient across the membrane, as required by the MD process. Isosteric heats of adsorption of water and toluene were calculated for the first time for a MD ceramic material, showing the transition from the hydrophilic heterogeneous surface of the starting SiC to a non-hydrophilic surface after modification.

Direct contact membrane distillation tests did not reveal defects in the modified membrane. The membrane had lower water permeability than the traditional hollow fibres polymeric modules, but the permeability might be increased by increasing pore size, reducing the thickness of channel walls, or considering different types of geometries. Despite that, the new membrane achieved nearly 100% rejection for NaCl, ibuprofen, and caffeine while it was permeable to water. The ability of the membrane to retain 2,4-dichlorophenol was curbed by the volatility of this compound. Membrane permeability and selectivity were stable, which indicated no pore wetting over the 8 h filtration experiments. Hence, both the method of membrane preparation and surface characterization reported here offer new bases for the development of robust ceramic MD membranes.

## Figures and Tables

**Table 1:** EDX analysis and contact angle of water droplets for a flat-sheet membrane, measured before (SiC-*fil*) and after modification (SiC-*fob*). Contact angle measurements were repeated after soaking the modified membranes in boiling water for one day and three days.

	Atom%				Water Contact Angle		
	Si	C	O	F	wet sample		
					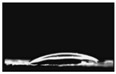		
SiC-*fil*	36.6	57.2	6.2	0.0	31.5° ± 3.7°		
					after modification	1 day in H_2_O—100 °C	3 days in H_2_O—100 °C
					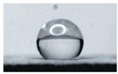	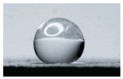	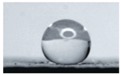
SiC-*fob*	36.6	57.0	5.6	1.5	143.2° ± 0.5°	146.8° ± 0.7°	148.9° ± 1.4°

## Supplementary Materials

The following are available online at https://www.mdpi.com/2079-4991/9/8/1159/s1.
